# Swallowing Characteristics and Water Swallow Capacity in Patients with Parkinsonism

**DOI:** 10.1007/s00455-024-10685-3

**Published:** 2024-05-04

**Authors:** Per Martell, Örjan Skogar, Liza Bergström

**Affiliations:** 1 Department of Speech and Language Pathology, Region Halland, 30185 Halmstad, Sweden; 2 Logopedics, Phoniatrics and Audiology, Department of Clinical Sciences, Lund University, Lund, Sweden; 3 Institute of Neuroscience and Physiology, Department of Health and Rehabilitation, Speech and Language Pathology Unit, Sahlgrenska Academy, University of Gothenburg, Gothenburg, Sweden; 4 Remeo Stockholm, Torsten Levenstams väg 8, SE-128 64 Stockholm, Sweden; 5 Department of Clinical Sciences, Division of Neurology, Karolinska Institute, Danderyd University Hospital, SE-182 88 Stockholm, Sweden; 6 Faculty of Medicine, University of Lund, Lund, Sweden

**Keywords:** Dysphagia, FEES, Aspiration, Timed water swallow test, Parkinsonism

## Abstract

Prevalence and characteristics of dysphagia (including aspiration) in patients with parkinsonism is variable, depending on type of assessment, diagnosis, disease stage and duration. The aim of this study was to further evaluate dysphagia characteristics in patients with different types of parkinsonism with both instrumental (Flexible Endoscopic Evaluation of Swallowing, *FEES*) and non-instrumental (Timed Water Swallow Test, *TWST*) assessments. Swallowing characteristics in 74 patients with parkinsonism were prospectively assessed using FEES and TWST. Statistics employed were (a) Spearman rank correlation to measure correlation between dysphagia results and Parkinson subtypes, disease severity and duration and (b) the non-parametric tests Mann Whitney U and Kruskal Wallis to measure difference between groups. Dysphagia was common, with 50 (67.6%) of the patients demonstrating a mild-severe Dysphagia Outcome Severity Scale (DOSS, level 1–5). During FEES, 42% aspirated and 68% of these had silent aspiration. Aspiration was seen more frequently with increased disease severity as per Hoehn and Yahr (H&Y) (*r* = .459, *p* = < 0.001) and disease duration (*r* = .269, *p* = .021). Thin liquid (IDDSI level 0) was the most common consistency to aspirate, and the frequency of aspiration decreased with thicker liquids. Dysphagia and aspiration are common in all subgroups of parkinsonism and seen in early stages of H&Y and within the first year of disease duration. Hence, it is recommended that these patients are evaluated early for optimal management and to avoid aspiration-related complications.

## Introduction

Parkinsonism is a degenerative disease characterized by a combination of key symptoms such as bradykinesia, tremor, rigidity and/or postural disturbances. It includes diagnoses such as primary idiopathic Parkinson’s disease (IPD), secondary parkinsonism (SP) due to vascular disease or neuroleptic exposures, and also atypical forms (AP) such as progressive supranuclear palsy (PSP), multiple system atrophy (MSA) and corticobasal degeneration (CBD) [1, 2]. IPD is a neurological impairment with a prevalence of 100–200 per 100 000 and an incidence of 15 per 100 000 per year. Progressive supranuclear palsy, MSA and CBD are less common with a reported prevalence ranging from 0.97 to 39.3 per 100 000 and an incidence from 0.29 to 3.8 per 100 000. The prevalence and incidence increase with age, within all subgroups of parkinsonism [3–7].

People with parkinsonism experience debilitating motor symptoms which can functionally impact all movements in everyday activities. Although these impairments and life impacts are well known, a lesser-known symptom is that of dysphagia (i.e., swallowing difficulties). Dysphagia, if not managed appropriately, may result in dehydration, malnutrition and complications such as aspiration pneumonia and death [8, 9]. Apart from the negative physical and health impacts, dysphagia has also shown to reduce health related quality of life (HRQoL) and may lead to social isolation [10]. Medical complications from dysphagia not only include poor oral intake (dehydration/malnutrition) but also respiratory infections such as aspiration pneumonia. One of the leading causes of death in parkinsonism is aspiration pneumonia, and the risk of death secondary to pneumonia is six times greater for patients with parkinsonism than compared to aged-matched controls [11].

Dysphagia in parkinsonism has been reported to be between 11 and 100%, depending on the patient cohort studied and the methodology used to identify/diagnose dysphagia [12]. Dysphagia may be apparent in any or all of the three phases of swallowing; the oral, pharyngeal and esophageal stages, and may be characterized by (a) an impaired ability to transport the bolus efficiently through these swallowing stages and/or (b) a poor airway protection during the swallow [13, 14]. A range of specific Parkinson-related dysphagia symptoms have been reported in the literature; oral components with repetitive pump movements with tongue, oral residue and premature spillage; pharyngeal components with pharyngeal residue, sensory deficits and reduced rate of spontaneous swallows; esophageal components with hypomotility and spasm of the esophagus [15]. Biomechanical change in the swallowing apparatus has also been reported with oropharyngeal bradykinesia, decreased epiglottic rotation, decreased pharyngeal constriction and reduced anterior hyoid movement [16, 17]. Events of laryngeal penetration and tracheal aspiration have been shown to range from 45.5 to 78.8% in patients with IPD, 58–90% in MSA and 30–67% in PSP [12, 18, 19].

Studies report that approximately 20% of IPD patients with dysphagia present with no cough response when they aspirate food or fluids into the trachea, a phenomenon known as silent aspiration [20]. In patients with IPD, silent aspiration increases with disease duration and has been shown to be a significant risk factor for respiratory infection, with an odds ratio of 9.75 to develop respiratory infection compared to non-aspirating IPD patients [21].

Evidently, the literature reports a wide range of dysphagia and aspiration characteristics and prevalence figures in parkinsonism. Objectively measured dysphagia has shown higher prevalence than subjective ratings (patient reported), which suggest that subjective ratings may not accurately detect dysphagia [22].

Dysphagia assessment requires the clinician to have a thorough knowledge of a patient’s case history, the underlying disease and expected swallow characteristics, particularly if access to instrumental assessment is lacking. Pflug et al. examined the rate of aspiration of different bolus types in patients with IPD, and identified that aspiration of water was more common than aspiration of bread and biscuit as detected via Flexible Endoscopic Evaluation of Swallowing (FEES) [20]. Miles et al. reported, in a cohort of patients with mixed etiology, that silent aspiration was more often present for thickened bolus in comparison to thin water bolus [23]. Swallowing capacity in patients with IPD has been shown to be decreased compared with healthy controls [24, 25]. Swallowing capacity is assessed as a quantifiable measurement using the Timed Water Swallow Test (TWST) and can be used to evaluate progress of swallow efficiency over time [26, 27].

To date, data regarding dysphagia and aspiration characteristics and prevalence is variable, depending on type of assessment, Parkinson’s etiology, disease stage and duration. Therefore, the current study aimed to investigate and further describe:

1. Dysphagia outcomes and characteristics of patients with different (a) parkinsonism diagnoses, (b) parkinsonism severity and (c) disease duration as assessed via Flexible Endoscopic Evaluation of Swallowing (FEES) and Timed Water Swallow Test (swallow capacity and efficiency).
2. Aspiration status (including silent aspiration) as per different liquid and food consistencies.

## Materials and Methods

This study is part of a larger research project and was conducted in accordance with the Declaration of Helsinki. Ethical approval was granted by the Lund Ethical Committee (registration number 2015/805). All participants provided informed and written consent independently or by proxy, prior to study inclusion.

### Study Setting and Participants

Data from the current study is part of a larger research project which was prospectively collected at a large regional hospital in Halland County, Sweden, during a three-month period in 2016, and a five-month period in 2018 (data collection period dependent on resource constraints). In the current study, all patients with confirmed parkinsonism who were referred to the speech pathology service for a speech and/or swallow evaluation by their neurologist were invited to participate in this study. Within the Neurology department it is common practice for the majority of patients with parkinsonism to be referred to the speech and language department for a speech, language and/or swallowing evaluation. A medical chart review (i.e., lab values, radiology, assessments) of each patient was made by an external neurologist to ensure the correct diagnosis and severity of disease as per Hoehn and Yahr [28]. The included patients were asked to take their medications as usual.

### Study Procedure

#### Assessment of Dysphagia Outcomes and Characteristics

Dysphagia outcomes and characteristics of patients with parkinsonism were assessed via FEES and TWST. All patients completed these two swallow assessments within a 1-hour time frame, except two, who were inpatients prioritized for other investigations. For these two patients both assessments were then completed within 24 h. FEES was completed first, prior to the TWST. Assessments were conducted by the same SLP with > 7 years’ experience in FEES and dysphagia management.

### Flexible Endoscopic Evaluation of Swallowing (FEES)

The FEES was conducted using a Karl Storz Telepack with a rhino-laryngoscope of 3.7 mm in outer diameter, with patients sitting in an examination chair or in an upright position in bed. The examination was performed without topical anesthesia. Each examination started with an evaluation of pharyngeal and laryngeal secretion pooling with the Secretion Severity Scale (SSS) [29], and an evaluation of pharyngeal and laryngeal structures and movements as per Langmore [30]. The patients were given food and fluids colored with blue food dye. The protocol included teaspoons (5 ml), sips and sequential drinking of the International Dysphagia Diet Standardisation Initiative (IDDSI) levels 0 and 2, a teaspoon and a tablespoon (15 ml) of IDDSI level 4 and bites of a “Mariekex” cracker (IDDSI level 7). The protocol was modified as necessary for patient safety. All patient’s FEES-examinations (*n* = 74) were rated using the Dysphagia Outcome Severity Scale (DOSS) [31], the Functional Oral Intake Scale (FOIS) [32] and the 8-point penetration-aspiration scale (PAS) to evaluate airway safety [33, 34] on site for optimal patient care. The FEES-recordings were later de-identified, randomized and stored for blinded re-evaluation 2–31 months later. The highest PAS score for every bolus was recorded. Trace amounts of aspiration was defined as less than 10% of bolus volume being ejected below the vocal folds resulting in a thin coating on the upper tracheal wall [35]. FEES ratings occurred using one SLP with > 7 years’ experience, as per above.

### Timed Water Swallow Test (TWST)

The TWST was performed as described by Hughes & Wiles [27]. The patient was instructed to drink 100 ml of water as quickly and comfortably as possible. A stopwatch was used and started the moment the water touched the bottom lip and stopped when the larynx was back at rest after the last swallow. The number of laryngeal elevations was used to count the number of swallows. Signs of coughing during and coughing or a wet voice after the test was noted. Total time, number of swallows and total amount of water were recorded to measure average swallow capacity (ml/sec), volume per swallow (ml/swallow) and time per swallow (seconds/swallow). If the patient couldn’t finish the total amount the residual amount was calculated and subtracted from 100 ml to get the total amount of water consumed.

### Statistical Analysis

Descriptive statistics were calculated, and statistical analyses were completed using SPSS v. 28 software. The current study’s power was calculated to be 85% with a sample size of *n* = 74 (alpha = 0.05), based on a dysphagia prevalence of 50% - considering the varying reports of dysphagia (11–100%) within the PD literature.

The mean, median, min and max were extracted for descriptive purposes. Due to non-normally distributed data the non-parametric test Mann Whitney U test was used to compare results between two groups. The non-parametric test Kruskal Wallis with post hoc pairwise comparison Bonferroni correction of *p*-values was used to compare results of three or more groups. The Spearman rho correlation was used to measure the strength of association between two variables. A p-value of < 0.05 was considered significant. For group comparisons, participants were divided into groups according to diagnosis, disease severity (H&Y) and disease duration. Subjects with the diagnoses of MSA and PSP were merged into one group (AP) for statistical analyses due to the small number of subjects in each group. The group comparison analyses were made for the variables of DOSS, FOIS, PAS, SSS and TWST (swallow capacity, volume per swallow, time per swallow, total time). Participants were also divided into groups according to gender, DOSS, FOIS and PAS for analyses and comparison of TWST results. PAS was categorized into three groups of (a) normal: PAS 1–2, (b) penetration: PAS 3–5 and (c) aspiration: PAS 6–8. H&Y, DOSS and FOIS were categorized into three subgroups based on severity levels ranging from normal to more severe levels of disability. Disease duration was divided into three groups: (a) 0–5 y, (b) 6–10 y and (c) > 10 y. No assumptions were made about missing data.

## Results

### Participants

In total, 76 patients were referred for evaluation, however two patients refused FEES, leaving a total of 74 included in this study cohort. Patient demographics are presented in Table 1. A significant difference between the diagnoses was evident in disease severity as per H&Y (*H* = 10.3, *p* = .006), disease duration (*H* = 6.8, *p* = .033) and the intake of levodopa (*H* = 13.0, *p* = .001). A post hoc Bonferroni pairwise comparison test showed a significant difference between (1) IPD - SP for disease duration, and (2) between IPD - AP, and SP - AP for disease severity and levels of Levodopa.

**Table 1:** Patient demographics

	IPD	SP	AP	Total	p-value^a^
Number of patients	41 (56%)	23 (31%)	10 (13%)	74 (100%)	–
Gender M/F (n)	24 / 17	18 / 5	6 / 4	48 / 26	0.273
Age (years)	72.8 ± 8.3	76.1 ± 7.3	69.8 ± 8.7	73.3 ± 8.2	0.114
Age (min – max)	50–86	61–87	58–83	50–87	–
H&Y stage 1 (n)	2 (5%)	0	0	2 (3%)	–
H&Y stage 2 (n)	15 (37%)	7 (30%)	0	22 (30%)	–
H&Y stage 3 (n)	10 (24%)	7 (30%)	2 (20%)	19 (26%)	–
H&Y stage 4 (n)	7 (17%)	6 (26%)	2 (20%)	15 (20%)	–
H&Y stage 5 (n)	7 (17%)	3 (13%)	6 (60%)	16 (22%)	–
H&Y (median)	3	3	5	3	0.006*
Disease duration (years)	9.9 ± 6.3	5.9 ± 4.4	7.7 ± 3.8	8.4 ± 5.7	0.033*
Disease duration (min – max)	1–23	1–18	3–13	1–23	–
Levodopa (mg/day)	566 ± 283	574 ± 272	205 ± 271	519 ± 301	0.001**
Levodopa (mg/day min-max)	100–1500	200–1050	0–850	0–1500	–

Values are mean ± standard deviation unless otherwise indicated

*n* number; *IPD* idiopathic Parkinson’s disease; *SP* secondary parkinsonism; *AP* atypical parkinsonism; *H&Y* Hoehn & Yahr

*p= < 0.05; **p= < 0.005

^a^Kruskal−Wallis*H*test

### (1a) Dysphagia Outcomes as per Parkinson Diagnosis

The grade of dysphagia was evaluated with DOSS and FOIS. See Table 2 for dysphagia grade, SSS and PAS scores for different diagnoses. There was no significant difference for diagnoses and outcomes on DOSS, SSS and PAS, however, a post hoc Bonferroni pairwise comparison test showed a significant difference between patients with IPD and AP when rated using FOIS (*H* = 6.7, *p* = .035).

**Table 2:** Dysphagia outcomes, aspiration and TWST results as per Parkinson diagnosis

	Diagnosis				
FEES results	IPD (*n* 41)	SP (*n* 23)	AP (*n* 10)	Total (*n* 74)	p-value^a^
DOSS	5	5	4	5	0.051
DOSS (min – max)	2–7	1–6	1–5	1–7	–
FOIS	6	5	5	5.5	0.035*
FOIS (min – max)	2–7	1–7	1–6	1–7	–
SSS	1	1	2	1	0.128
SSS (min – max)	0–3	0–3	0–3	0–3	–
PAS	3	3	7.5	4	0.174
Silent aspiration (PAS 8)	*n =* 9 (22%)	*n =* 7 (30%)	*n* = 5 (50%)	*n =* 21 (28%)	
Aspiration events (PAS 6–8)	*n =* 15 (37%)	*n =* 10 (43%)	*n* = 6 (60%)	*n =* 31 (42%)	
Penetration (PAS 3–5)	*n =* 12 (29%)	*n =* 4 (17%)	*n* = 3 (30%)	*n =* 19 (26%)	
Normal (PAS 1–2)	*n =* 14 (34%)	*n =* 9 (39%)	*n* = 1 (10%)	*n =* 24 (32%)	
TWST results	IPD (*n* 38)	SP (*n* 20)	AP (*n* 8)	Total (*n* 66)	
Swallow capacity (ml/s)	8.4 ± 5.4	8 ± 4.5	4.9 ± 4.7	7.9 ± 5.1	0.170
Volume per swallow (ml/sw)	12.4 ± 5.3	12.6 ± 5.6	12.6 ± 9.7	12.5 ± 5.9	0.660
Time per swallow (s/sw)	2.1 ± 1.7	2 ± 1.4	4.4 ± 3.2	2.4 ± 2.0	0.064
TWST 100 ml	IPD (*n* 34)	SP (*n* 20)	AP (*n* 7)	Total (*n* 61)	
Total time (seconds)	19.8 ± 19.5	17.3 ± 12.1	34.5 ± 31.8	20.7 ± 19.6	0.306

Values are median for ordinal data (DOSS, FOIS, SSS, PAS) and mean ± standard deviation for interval data (TWST results) unless otherwise indicated

*n* number; *TWST* Timed water swallow test; *FEES* Flexible endoscopic evaluation of swallowing; *IPD* idiopathic Parkinson’s disease; *SP* secondary parkinsonism; *AP* atypical parkinsonism; *DOSS* Dysphagia Outcome Severity Scale; *FOIS* Functional Oral Intake Scale; *SSS* Secretion Severity Scale; *PAS* Penetration-Aspiration scale; *ml/s* milliliter per second; *ml/sw* milliliter per swallow; *s/sw* seconds per swallow

*p= < 0.05; **p= < 0.005

a=Kruskal−Wallis*H*test

Of the 74 patients, 67.6% (*n* = 50) were graded as dysphagic as per DOSS level 1–5. During FEES 31/74 (42%) of the patients had aspiration events, and 21/31 (68%) of the aspirators had silent aspiration (PAS 8) at least once during the examination. For those patients who silently aspirated, 11/21 (52%) were rated to have trace aspiration, while eight of these patients had a cough response (PAS 7) when a larger amount was aspirated.

Regarding secretion ratings, there was no significant difference across diagnoses (*H* = 4.1, *p* = .128). There was, however, a significant difference between aspirators and non-aspirators (*U* = 1077, *p* = < 0.001) across diagnoses. A moderate correlation was found for PAS score and SSS (*r* = .555, *p* = < 0.001). Within the group with a normal score of SSS (0), 15% was aspirators, while within the group with a severe SSS score of 3, 89% was aspirators.

### (1b) Dysphagia Outcomes as per Parkinson Severity

See Table 3 for dysphagia grade, SSS and PAS scores for different levels of H&Y. There was a significant difference seen for DOSS (*H* = 21.7, *p* = < 0.001), FOIS (*H* = 24.9, *p* = < 0.001), SSS (*H* = 8.1, *p* = .017) and PAS (*H* = 12.7, *p* = .002) scores between different stages of H&Y. A post hoc Bonferroni pairwise comparison test showed a significant difference between patients with H&Y stage 1–3 and 4–5 for DOSS, FOIS and SSS and a difference between H&Y stage 1–2 and 4–5 for PAS. A statistically significant moderate, negative correlation between level of DOSS, FOIS and stage of H&Y (*r* = − .501, *p* = < 0.001 and *r* = − .516, *p* = < 0.001, respectively) was found.

**Table 3:** Dysphagia outcomes, aspiration and TWST results as per different stages of Hoehn and Yahr and disease duration

	H&Y stage				Disease duration (years)			
FEES results	1–2 (*n* 24)	3 (*n* 19)	4–5 (*n* 31)	p-value^a^	0–5 (*n* 31)	6–10 (*n* 22)	> 10 (*n* 21)	p-value^a^
DOSS	5.5	6	4	0.001**	5	5	4	0.250
FOIS	7	7	5	0.001**	6	5	5	0.113
SSS	0.5	1	1	0.017*	1	1	1	0.360
PAS	2	3	7	0.002**	3	6	7	0.240
Silent aspiration (PAS 8)	*n* = 3 (13%)	*n* = 5 (26%)	*n* = 13 (42%)		*n* = 5 (16%)	*n* = 8 (36%)	*n* = 8 (38%)	
Aspiration events (PAS 6–8)	*n* = 4 (17%)	*n* = 7 (37%)	*n* = 20 (64%)		*n* = 8 (26%)	*n* = 11 (50%)	*n* = 12 (57%)	
Penetration (PAS 3–5)	*n* = 7 (29%)	*n* = 4 (21%)	*n* = 8 (26%)		*n* = 13 (42%)	*n* = 4 (18%)	*n* = 2 (10%)	
Normal (PAS 1–2)	*n* = 13 (54%)	*n* = 8 (42%)	*n* = 3 (10%)		*n* = 10 (32%)	*n* = 7 (32%)	*n* = 7 (33%)	
TWST results	1–2 (*n* 24)	3 (*n* 18)	4–5 (*n* 24)		0–5 (*n* 28)	6–10 (*n* 21)	> 10 (*n* 17)	
Swallow capacity (ml/s)	10.1 ± 4.2	7.8 ± 5.2	5.7 ± 5.0	0.003**	9.4 ± 4.6	6.7 ± 4.9	6.8 ± 5.7	0.058
Volume per swallow (ml/sw)	13.8 ± 5.0	13.9 ± 7.4	10.1 ± 5.1	0.023*	14.6 ± 6.1	11.3 ± 6	10.5 ± 4.5	0.031*
Time per swallow (s/sw)	1.5 ± 0.5	2.4 ± 1.4	3.3 ± 2.8	0.012*	1.9 ± 1.2	2.5 ± 2.3	2.8 ± 2.4	0.392
TWST 100 ml	1–2 (*n* 24)	3 (*n* 17)	4–5 (*n* 20)		0–5 (*n* 27)	5–10 (*n* 20)	> 10 (*n* 14)	
Total time (seconds)	12.0 ± 6.6	18.4 ± 12.8	33.0 ± 27.6	0.018*	14.0 ± 10.8	26.0 ± 22.0	26.0 ± 25.8	0.069

Values are median for ordinal data (DOSS, FOIS, SSS, PAS) and mean ± standard deviation for interval data (TWST results) unless otherwise indicated

*n* number; *TWST* Timed water swallow test; *FEES* Flexible endoscopic evaluation of swallowing; *DOSS* Dysphagia Outcome Severity Scale; *FOIS* Functional Oral Intake Scale; *SSS* Secretion Severity Scale; *PAS* Penetration-Aspiration scale; *ml/s* milliliter per second; *ml/sw* milliliter per swallow; *s/sw* seconds per swallow

*p= < 0.05; **p= < 0.005

a=Kruskal−Wallis*H*test

Aspiration was seen more frequently with increased stage of H&Y. There was a statistically significant moderate, positive correlation between level of PAS and stage of H&Y (*r* = .459, *p* = < 0.001). Even if aspiration was seen more frequently with increased stage of H&Y, it is evident that severe levels of PAS also occurred in early stages of H&Y, where 3/22 (14%) patients with H&Y stage 2 had silent aspiration. A normal PAS score of 1–2 was not seen for any of the patients in H&Y stage 5. A statistically significant weak, positive correlation between SSS and stage of H&Y was found (*r* = .350, *p* = .002).

### (1c) Dysphagia Outcomes as per Parkinson Disease Duration and Levodopa Intake

A statistically significant weak negative correlation for disease duration and both DOSS (*r* = − .245, *p* = .035) and FOIS (*r* = − .281, *p* = .015) was found. There was a weak, positive correlation between PAS score and disease duration, which was statistically significant (*r* = .269, *p* = .021). Silent aspiration occurred in 1 of 6 patients (17%) with a disease duration of only 1 year. There was no correlation found between PAS scores and Levodopa intake (*r* = − .004, *p* = .975), or age (*r* = .035, *p* = .770).

### Results of the Timed Water Swallow Test

The TWST test was performed in 66/74 patients. Eight patients did not perform the TWST due to patient safety. Of the eight excluded patients five had major silent aspiration and three major audible aspiration during FEES. Of the 66 patients that took the TWST, 92.4% (*n* = 61) finished the total amount of 100 ml. A significant difference was found between patients with aspiration and non-aspirators (*U* = 185, *p* = .001). See tables for overall cohort TWST results: diagnosis (Table 2), H&Y stage (Table 3) and gender, DOSS, FOIS and PAS (Table 4).

**Table 4:** TWST results for gender, DOSS, FOIS and PAS

	Gender				DOSS				FOIS				PAS		
	Male (*n* 41)	Female (*n* 25)	Total (*n* 66)	p-value^a^	1–2 (*n* 7)	3–5 (*n* 35)	6–7 (*n* 24)	p-value^b^	1–3 (*n* 8)	4–6 (*n* 30)	7 (*n* 28)	p-value^b^	PAS 1–5 (*n* 43)	PAS 6–8 (*n* 23)	p-value ^b^
Swallow capacity (ml/s)	8.5 ± 4.6	6.8 ± 5.8	7.9 ± 5	0.128	2.5 ± 2.1	7.4 ± 4.8	10.1 ± 4.9	0.001**	3.1 ± 2.6	6.7 ± 4.4	10.5 ± 4.9	0.001**	9.6 ± 5.1	4.7 ± 3.3	0.001**
Volume per swallow (ml/sw)	12.7 ± 5.8	12.2 ± 6.2	12.5 ± 5.9	0.457	7.8 ± 4.0	12.5 ± 6.1	13.9 ± 5.5	0.032*	8.1 ± 3.8	12.0 ± 6.0	14.2 ± 5.7	0.015*	13.6 ± 5.6	10.4 ± 6.1	0.011*
Time per swallow (s/sw)	1.9 ± 1.6	3.1 ± 2.4	2.4 ± 2	0.016*	3.9 ± 2.2	2.6 ± 2.2	1.6 ± 0.9	0.002**	3.6 ± 2.2	2.8 ± 2.4	1.5 ± 0.8	0.001**	2.0 ± 2	3 ± 1.8	0.001**
TWST 100 ml	Male (*n* 39)	Female (*n* 22)	Total (*n* 61)	p-value^a^	1–2 (*n* 5)	3–5 (*n* 32)	6–7 (*n* 24)	p-value^b^	1–3 (*n* 5	4–6 (*n* 28)	7 (*n* 28)	p-value^b^	PAS 1–5 (*n* 43)	PAS 6–8 (*n* 18)	p-value ^b^
Total time (seconds)	17.2 ± 16.5	26.9 ± 23.2	20.7 ± 19.6	0.270	45.6 ± 33.6	22.7 ± 20.0	12.7 ± 7.7	0.010*	45.6 ± 33.6	24.8 ± 20.6	12.1 ± 7.3	0.002**	16.7 ± 16.6	30.2 ± 23.2	0.001**

Values are mean ± standard deviation unless otherwise indicated

*n* number; *TWST* Timed water swallow test; *DOSS* Dysphagia Outcome Severity Scale; *FOIS* Functional Oral Intake Scale; *PAS* Penetration-Aspiration scale; *ml/s* milliliter per second; *ml/sw* milliliter per swallow; *s/sw* seconds per swallow

*p < .05.**p < .005

^a^Mann Whitney

^b^Kruskal−Wallis*H*test

### (2) Aspiration Status as per Different Liquid and Food Consistencies

The prevalence of aspiration to specific bolus types differed. The rate of aspiration was significantly higher with thin liquid (IDDSI level 0) compared to thicker liquid (IDDSI level 2) and pudding (IDDSI level 4) as well as for biscuit (IDDSI level 7) (*H =* 26.3, *p* = < 0.001). See Table 5 for information on PAS scores for different bolus types. For those patients with aspiration events, 17/31 (55%) had trials of all IDDSI levels. Of these patients, 12/17 (70.6%) had aspiration for one consistency only and 5/17 (29.4%) had for more than one. Within-participant differences in responsiveness to aspiration were not observed across consistencies for those patients.

**Table 5:** PAS scores for different bolus types as per IDDSI

	IDDSI 0 (n 70)	IDDSI 2 (n 68)	IDDSI 4 (n 72)	IDDSI 7 (n 59)	p-value^a^
PAS	3	1	1	1	< 0.001**
Silent aspiration (PAS 8)	*n =* 18 (26%)	*n =* 8 (12%)	*n =* 2 (3%)	*n =* 0	
Aspiration events (PAS 6–8)	*n =* 27 (39%)	*n =* 8 (12%)	*n =* 4 (6%)	*n =* 1 (2%)	
Penetration (PAS 3–5)	*n =* 17 (24%)	*n =* 17 (25%)	*n =* 17 (24%)	*n =* 7 (12%)	
Normal (PAS 1–2)	*n =* 26 (37%)	*n =* 43 (63%)	*n =* 51 (71%)	*n =* 51 (86%)	

Values are median unless otherwise indicated

*n* number; *PAS* Penetration-Aspiration scale; *IDDSI* The International Dysphagia Diet Standardisation Initiative

**p < .005

^b^Kruskal−Wallis*H*test

## Discussion

The goal of the current study was to investigate dysphagia characteristics and swallow capacity in a mixed cohort of patients with parkinsonism with the aim to provide clinically relevant data for future dysphagia management. Results demonstrate that penetration and aspiration is a common phenomenon among patients with different types of parkinsonism, regardless of severity as per H&Y, or disease duration. Most patients had aspiration events with IDDSI level 0 versus thicker boluses and there was no difference in patient’s cough response when aspiration occurred across consistencies. Patients with more severe parkinsonism presented with worse swallow capacity/efficiency, however there was no significant difference across subtypes of parkinsonism. Interestingly, however, dysphagia severity (grade of dysphagia, as per DOSS) did not differ significantly between different diagnoses. A significant difference and deterioration of dysphagia was evidenced only between different H&Y stages. These results, along with aspiration and TWST outcomes will be further discussed below in terms of clinical application and international literature.

### (1a) Dysphagia Outcomes, Aspiration & TWST as per Parkinson Diagnosis

The prevalence of dysphagia (67.6%) with subsequent penetration and aspiration in the present study (68% and 42%, respectively) has similarities and incongruencies to earlier research [12, 18, 19, 23]. Regarding dysphagia severity, results from the current study suggest a significant difference between different Parkinson diagnoses as per FOIS, however not per DOSS. Initially, this was considered to be an interesting finding, since according to Passos [36] FOIS and DOSS are strongly correlated, i.e., a high score on FOIS correlates to a high score on DOSS. Upon closer inspection of the data, perhaps the reason that there was a significant difference between groups with FOIS, and not DOSS, is due to this study’s sample size. For example, DOSS results were approaching significance (*p* = .051) and may have reached significance with a larger sample size.

In terms of penetration and aspiration characteristics, studies by Lee et al. [18], Luchesi et al. [12], and Warnecke et al. [19] demonstrated similar outcomes to the current study, with high dysphagia, penetration and aspiration results. No significant difference in prevalence of PAS score between diagnoses was demonstrated in the current study, although there was a higher PAS score trend for the AP-group in comparison with the IPD and SP cohorts.

Results of the current study, however, are somewhat incongruent with other penetration-aspiration reports [23]. Regarding aspiration, Miles et al. [23], demonstrated that the cough response to aspiration is inconsistent across bolus volumes and viscosities. In their study, a higher prevalence of silent aspiration was found with thick liquids compared to thin liquids. This is in contrast to the results of the current study, where the patient’s cough response to aspiration, was not significantly different across consistencies for patients who had aspiration with more than one consistency. Of note, though, is that even though the total number of aspirators in the current study was high, only a small number of patients aspirated on more than one consistency.

In terms of the TWST results (the capacity for drinking a large amount of water quickly and effectively), the current study suggests that swallow capacity and efficiency seems to radically decrease when considering different aspects of parkinsonism. Overall, across all parkinsonian parameters (subtype, severity and duration of disease), the total mean time for drinking 100 ml water was almost three times longer, and capacity in ml/sec almost three times lower for patients compared to what has been reported on healthy Swedish adults [37]. This could be explained to some extent by the high prevalence of dysphagia in the studied cohort. However, when only including patients with a normal swallow function as per DOSS (score of 6 and 7), the total mean time of drinking 100 ml water was still almost double as to healthy aged-matched controls [37]. This difference could be an effect of bradykinesia, which is a key symptom in parkinsonism.

Regarding the swallow capacity parameters (ml/sec, ml/swallow, sec/swallow) and results between subtypes of parkinsonism, our results are similar to what has been reported by Kanna & Bhanu [24] who examined 100 patients with IPD with a water test of 150 ml. The current study included other parkinsonian diagnoses compared to Kanna & Bhanu who only included patients with IPD. Even so, the results are similar, indicating that the swallow capacity parameters may apply to any form of parkinsonism. This is evident by results from this study showing no significant difference in capacity parameters between the diagnoses included in the study. Data from the current study, might therefore be useful as a reference for what to expect with different subtypes of parkinsonism.

### (1b) Dysphagia Outcomes, Aspiration & TWST as per Parkinson Severity

In the current study, increased dysphagia severity as per DOSS and FOIS was seen with an increase in H&Y stage (*r* = − .501, *p* = < 0.001 and *r* = − .516, *p* = < 0.001, respectively). The impact of greater disease severity (increased level of H&Y) on prevalence of dysphagia is supported by other studies reporting similar findings [20, 38, 39]. Han et al. [39] used the “Swallowing disturbances Questionnaire” to evaluate the prevalence of dysphagia considering different levels of H&Y and found that 100% of the patients in H&Y 5 subjectively identified dysphagia, which correlates well with the objective findings from this study.

The results show that swallow safety as per PAS score is decreased by each stage of H&Y, and radically decreased for patients in H&Y stage 4 and 5 where 80% and 100% of patients, respectively, had either penetration or aspiration. Pflug et al. [20] examined 119 patients with Parkinson’s disease with FEES and found that the prevalence of penetration and aspiration tended to increase with increased severity of disease. Ding et al. [38] examined 116 patients with PD with VFSS and concluded that the risk of having dysphagia was 3.26 times higher for patients in higher H&Y stages compared to patients in lower staging.

Within the ESPEN guideline for clinical nutrition in neurology it has been recommended that screening for dysphagia occur from H&Y stage 3 and above, due to the high risk of aspiration [40]. In this study 17% of the patients with H&Y stage 1–2 had aspiration, and as many as 46% had either penetration or aspiration which suggests that a screening program from H&Y stage 1–2 would be advantageous for optimal patient management. This is also supported by the study by Pflug et al. [20] where 12% had aspiration (PAS score of 7 and 8) also in the earlier H&Y stage 2.

Regarding TWST (swallow capacity and efficiency), previous literature has shown significant correlation with Parkinson disease severity as per Unified Parkinson’s Disease Rating Scale and the H&Y scale [24]. In the study by Kanna & Bhanu, a decreased swallow performance was seen when the H&Y staging ≥ 3. Similarly, the present study also found that an increased H&Y level leads to a decreased swallow capacity, especially for patients with the most severe level of H&Y (5). In our study, the presence of aspiration had a significant impact on swallow capacity, and this might explain the decreased swallow capacity that is seen with increased H&Y level, where aspiration is seen more frequently. Patients with more severe parkinsonism also presented with a greater overall motor impairment and bradykinesia, which may also have negatively impacted the patient’s ability to drink quickly and efficiently. Results from the current study and previous literature suggest that a routine swallow screen would be of value for all H&Y stages of parkinsonism and could be incorporated as an initial screen in Neurology departments to early identify patients in need of a more comprehensive assessment.

### (1c) Dysphagia Outcomes, Aspiration & TWST as per Parkinson Duration

In this study, no significant difference was seen with dysphagia severity as per DOSS, FOIS or PAS scores when considering disease duration - dividing patients into three groups with duration of (a) 0–5 y, (b) 6–10 y and c) > 10 y. However, a weak correlation was seen for disease duration and dysphagia level (DOSS: *r* = − .245, *p* = .035; FOIS: *r* = − .281, *p* = .015) and penetration-aspiration status (PAS: *r* = .269, *p* = .021). This is congruent with the results by Cereda et al. [41] who demonstrated that disease duration was associated with increased levels of swallowing disturbances. Other studies, however, have suggested that dysphagia can be evident at any time during disease duration [15, 42]. In the present study, aspiration was seen in 1 of 6 (17%) patients with disease duration of less than one year, and Pflug et al. [20] had similar findings where 20% of patients with a disease duration of less than two years demonstrated aspiration. Again, this highlights the importance of early systematic screening in this patient population.

For TWST, a significant difference between groups for disease duration regarding volume per swallow, and a trend in decreased capacity and efficiency for ml per second and total drinking time was seen. These results are congruent with previous literature, where Kanna & Bhanu [24] showed a significant correlation for TWST and Parkinson disease duration, especially patients with IPD > 5 years had a decline in swallowing capacity. In terms of clinical application, data from this and other research supports the argument that patients with parkinsonism should not only be screened early, but also access regular dysphagia follow-up as time and disease severity progresses.

### (2) Aspiration Status as per Different Liquid and Food Consistencies

In the current study, water (IDDSI level 0) was found to be the bolus type with highest prevalence of penetration and aspiration (63%), compared to thicker liquids and solid bolus (14–37%). Pflug et al. [20] similarly found that water was the bolus type with highest prevalence of penetration and aspiration in patients with IPD. However, they did not compare water to other forms of thickened liquids, only to solid food. The high frequency of aspiration to water compared to thicker liquids can be explained by the different types of oropharyngeal swallowing abnormalities that has been shown to be present in patients with parkinsonism. Kim et al. [17] performed a two-dimensional motion analysis on patients with IPD and compared their swallow kinematics with healthy individuals. The most noticeable findings in patients with IPD was oropharyngeal bradykinesia, incoordination, decreased epiglottic rotation and reduced anterior hyoid bone movement, all of which can contribute to decreased swallow safety. The rationale for using thickened liquids is to slow the bolus flow to allow more time for laryngeal vestibule closure [43] which might explain the low rate of aspiration for thicker liquids in this population. This finding may have particular clinical significance in dysphagia management where bolus modification plays an important role to reduce aspiration events for some patients. Specifically for the parkinsonism population, it might be ideal to choose thicker liquids when taking oral medicine/Levodopa to minimize the risk of aspirating medication which could lead to serious medical adverse events.

The relationship between (i) accumulated secretions (SSS) in the pharynx and larynx, and (ii) laryngeal penetration/aspiration when eating/drinking has previously been shown to have a strong correlation [29, 44–46]. Kuo et al. [45] suggests that the SSS can be used to predict aspiration in patients with dysphagia. In their study patients with a SSS of 2 or above were shown to have an odds ratio of 13.6 of presenting with aspiration compared to patients with a lower SSS grade. The result in this study shows that when a patient with parkinsonism presents with a SSS score of 0, then 15% of the patients aspirated, compared to 89% of those patients who had a SSS score of 3. Secretion rating may hold the potential to be used as a quick clinical screen for increased risk of aspiration if performing a laryngeal mirror examination. This does not require expensive equipment and could be regularly performed by a nurse or neurologist when the patient with parkinsonism visits the Neurology department.

### Limitations and Future Directions

As with all research, the current study has its limitations. One limitation of the study is the referral process from the Neurology department. Even though the majority of patients with parkinsonism visiting the Neurology department are routinely referred to SLP services, there is no standardized protocol to ensure that all patients receive SLP screen or evaluation. This might have led to a skewness in the selection of participants, given a more symptomatic subset of included patients. For future research it would be valuable to include all patients visiting the Neurology department, and not just the subgroup that is referred to the SLP department.

Eight participants did not undergo the TWST test, since the FEES, which occurred prior to the TWST, showed events of major aspiration. Given this and that the eight participants were medically fragile with poor general condition, an ethical decision not to administer the TWST to these patients was made. A limitation is that this may have skewed the data, particularly in the swallow capacity parameters. Another limitation, is the lack of inter-rater reliability testing of the FEES results. All assessments were conducted by the same SLP often within one hour, however, to ensure rigorous methodology, the FEES reference test was recorded for later re-analysis. Recorded films from the FEES were de-identified, randomized and rated 2–31 months later. For future research an independent FEES assessor could be utilized to ensure a greater level of blinding and possibility of inter-rater reliability testing.

In terms of outcome measures, a limitation in this study is that DOSS was used as an outcome measure with FEES, though DOSS is not validated against FEES. Another limitation is that evaluation of pharyngeal residue has not been included in the analysis - a recommendation for future research. An additional consideration for future improvement is to perhaps use the New Zealand Secretion Scale (NZSS) [47], which considers a sensory aspect/response, rather than the Secretion Severity Scale which does not. The NZSS, however, had not been published at the time.

Finally, a general limitation of the study is the limited sample size for each of the parkinsonism subgroups that did not allow for subgroup statistical analyses.

## Conclusion

Dysphagia and aspiration, in particular silent aspiration, is a common finding in different types of parkinsonism. A high portion of the patients with parkinsonism in this study presented with penetration and aspiration regardless of diagnosis, disease duration and level of H&Y. Increased disease duration and level of H&Y seems to lead to higher prevalence of aspiration. Given that dysphagia and events of silent and audible aspiration are frequently seen early in this patient population’s disease duration and in the lower severity levels of H&Y, it is important to consider early systematic evaluation of all patients diagnosed with parkinsonism.
